# Supplementary material for: Assessing the health risk of living near composting facilities on lung health, fungal and bacterial disease in cystic fibrosis: a UK CF Registry study
Source: Environ Health. 2022 Dec 15;21:130. doi: 10.1186/s12940-022-00932-1 (PMC9753251; doi:10.1186/s12940-022-00932-1)
Supplement: Supplementary file 1 — Additional file 1: Appendix A. Allergic Bronchopulmonary Aspergillosis and Candida spp. [file 12940_2022_932_MOESM1_ESM.docx]

***Appendix A: Allergic Bronchopulmonary Aspergillosis and Candida spp.***

ABPA is defined using standard international diagnostic criteria, including acute or subacute clinical deterioration (cough, wheeze, exercise intolerance, exercise-induced asthma, change in pulmonary function, or increased sputum production) not attributable to another aetiology, total IgE > 500 IU/ml and a positive skin prick test for *Aspergillus* antigen (> 3 mm) or positive specific IgE for *A. fumigatus.* Also, either precipitating to *A. fumigatus* or *in vitro* demonstration of IgG antibodies to *A. fumigatus* or new or recent abnormalities on chest radiography (infiltrates or mucus plugging) or chest CT (characteristic changes) that have not cleared with antibiotics and standard physiotherapy^1^. The point prevalence of ABPA was 8.8% among PwCF living close to a PCS compared to 7.9% in those living farther away (p= 0.397).
