# Supplementary material for: Assessing the health risk of living near composting facilities on lung health, fungal and bacterial disease in cystic fibrosis: a UK CF Registry study
Source: Environ Health. 2022 Dec 15;21:130. doi: 10.1186/s12940-022-00932-1 (PMC9753251; doi:10.1186/s12940-022-00932-1)
Supplement: Supplementary file 2 — Additional file 2: Appendix B. Distance bands. [file 12940_2022_932_MOESM2_ESM.docx]

***Appendix B: Distance bands***

In line with previous studies^2-4^, we considered multiple distance bands around each PCS (Table S1). Analyses were conducted for each of the following distance bands (≤250m; 250-750m; 750m-1,5km; 1,5-4,0km and >4,0km). Only results for within and beyond 4km are presented due to the lack of statistical power due to small numbers of pwCF living in the smaller radii.
