# Supplementary material for: Assessing the health risk of living near composting facilities on lung health, fungal and bacterial disease in cystic fibrosis: a UK CF Registry study
Source: Environ Health. 2022 Dec 15;21:130. doi: 10.1186/s12940-022-00932-1 (PMC9753251; doi:10.1186/s12940-022-00932-1)
Supplement: Supplementary file 3 — Additional file 3: Appendix C. Rural-urban. [file 12940_2022_932_MOESM3_ESM.docx]

***Appendix C: Rural-urban***

In England and Wales, all urban major/ minor conurbations (population of 10,000 or more) and urban cities are classified into urban area^5^. Rural towns, fringes, villages, hamlets and isolated dwellings were classified as a rural area. We have extracted rural and urban areas classification from ONS postcodes directly based on residential postcode (<https://geoportal.statistics.gov.uk/>). In Scotland, rural areas are classified as an area with a settlement of fewer than 3,000 people and settlement of over 3,000 people is classified as urban. In Northern Ireland, the urban area is defined as an area with a population of 5,000 or more and an area with a population of less than 5,000 is classified as rural.
