# Supplementary material for: Assessing the health risk of living near composting facilities on lung health, fungal and bacterial disease in cystic fibrosis: a UK CF Registry study
Source: Environ Health. 2022 Dec 15;21:130. doi: 10.1186/s12940-022-00932-1 (PMC9753251; doi:10.1186/s12940-022-00932-1)
Supplement: Supplementary file 4 — Additional file 4: Table S1. Number of people with CF (pwCF) living within specific distances from a permitted composting site (PCS) in the UK in 2016. Table S2. Classification of CFTR genotypes with frequencies among pwCF (n=9,361) in the UK in 2016. Table S3. Linear regression analysis of the relationship between ppFEV1 and residential postcode distance from PCS among children and adults with CF in the UK in 2016. p-value <0.05 are shown in bold. [file 12940_2022_932_MOESM4_ESM.docx]

**Supplementary Tables**

**Table S1.** Number of people with CF (pwCF) living within specific distances from a permitted composting site (PCS) in the UK in 2016

| *Distance to a PCS* | *pwCF* | *%* |
| --- | --- | --- |
| 0-250 m | 0 | 0.0 |
| > 250 - ≤ 750 m | 18 | 0.2 |
| > 750 - ≤ 1.5 km | 89 | 1.0 |
| > 1,5 - ≤ 2.5 km | 260 | 2.8 |
| > 2.5 - ≤ 4.0 km | 649 | 6.9 |
| > 4.0 km | 8,345 | 89.1 |
| TOTAL | 9,361 | 100.0 |

**Table S2:** Classification of *CFTR* genotypes with frequencies among pwCF (n=9,361) in the UK in 2016

| SNO | Classification | Description | n | % |
| --- | --- | --- | --- | --- |
| 1 | **Homozygous F508del** | F508del on both alleles. | 4,603 | 49.2 |
| 2 | **F508del/ Minimal function (MF)** | F508del on one allele and any minimal function mutation on the other allele i.e., a mutation that produces either no protein or protein that does not respond to currently approved *CFTR* modulators, | 1,822 | 19.5 |
| 3 | **Residual function (RF)/other** | RF mutations include 2789+5G>A, 3272-26A>G, 3849+10kbC>T, 711+3A->G, A1067T, A455E, D110E, D110H, D1152H, D1270N, D579Gx, E193K, E56K, E831X, F1052V, F1074L, G1069R, K1060T, L206W, P67L, R1070Q, R1070W, R117C, R347H, R352Q, R74W, S945L and S977F  RF on one allele and any other mutation on the 2nd allele were counted in this group including RF/RF, F508del/RF, MF/RF, RF/Gating and RF/Other. This group also includes R117H. | 1,070 | 11.4 |
| 4 | **Gating (G)/any*** | G551D, G1244E, G1349D, G178R, G551S, S1251N, S1255P, S549N, or S549R. Gating on one allele and any other mutation on the 2nd allele including F508del/G, G/G, G/Other and MF/G but not RF/G (see above) | 583 | 6.2 |
| 5 | **MF/MF** | All genotypes with MF mutations on both alleles | 298 | 3.2 |
| 6 | **F508del/Other** | F508del on one allele and the second allele was either not identified or identified but classification into the above groups was not possible | 663 | 7.1 |
| 7 | **MF/other** | MF on one allele and the second allele was either not identified or identified but classification into the above groups was not possible | 146 | 1.6 |
| 8 | **Other/other** | On both alleles either the mutation was not identified, or it was identified but classification into the above categories was not possible. | 176 | 1.9 |

*****Excludes Residual

**Table S3:** Linear regression analysis of the relationship between ppFEV_1_ and residential postcode distance from PCS among children and adults with CF in the UK in 2016. p-value <0.05 are shown in bold

| **ppFEV_1_** | **Children (6 -15 years)**  (n=2,370) | | **Adult (16 - 86 years)**  (n=5,430) | |
| --- | --- | --- | --- | --- |
|  | ≤ 4 km  (n=222) | > 4 km  (n=2148) | ≤ 4 km  (n=644) | > 4 km  (n=4786) |
| **Unadjusted**  β (95%CI) | -0.75 (-2.45, 0.96) | Ref | -1.44 (-2.84, -0.04) | Ref |
| *p value* | 0.391 | | **0.044** | |
| AIC | 19297.9 | | 47438.5 | |
| **Adjusted model I**  β (95%CI) | -0.90 (-2.54, 0.74) | Ref | -1.47 (-2.80, -0.14) | Ref |
| *p value* | 0.282 | | **0.031** | |
| AIC | 19118.11 | | 46929.3 | |
| **Adjusted model II**  β (95%CI) | -1.11 (-2.69, 0.47) | Ref | -1.50 (-2.78, -0.22) | Ref |
| *p value* | 0.168 | | **0.022** | |
| AIC | 18935.0 | | 46535.8 | |
| **Adjusted model III**  β (95%CI) | -1.06 (-2.63, 0.51) | Ref | -1.34 (-2.60, -0.10) | Ref |
| *p value* | 0.184 | | **0.034** | |
| AIC | 18910.4 | | 46185.4 | |
| **Adjusted model IV**  β (95%CI) | -1.00 (-2.56, 0.57) | Ref | -1.20 (-2.42, 0.03) | Ref |
| *p value* | 0.211 | | 0.056 | |
| AIC | 18896.5 | | 46062.6 | |
| **Adjusted model V**  β (95%CI) | -0.71 (-2.8, 0.87) | Ref | -1.05 (-2.28, 0.17) | Ref |
| *p value* | 0.377 | | 0.092 | |
| AIC | 18887.7 | | 46041.3 | |
| **Adjusted model VI**  β (95%CI) | -0.74 (-2.31, 0.83) | Ref | -1.07 (-2.29, 0.16) | Ref |
| *p value* | 0.343 | | 0.088 | |
| AIC | 18887.6 | | 46040.6 | |

ppFEV_1_ = percent predicted FEV_1_; CF = Cystic fibrosis; PCS = permitted composting site

| **Children**  Model I adjusted for BMI percentile  Model II adjusted for BMI percentile and age,  Model III adjusted for BMI percentile, age and mutation  Model IV adjusted for BMI percentile, age, mutation and *P. aeruginosa*  Model V adjusted for BMI percentile, age, mutation and *P. aeruginosa* and deprivation  Model VI adjusted for BMI percentile, age, mutation and *P. aeruginosa,* deprivation and sex | **Adults**  Model I adjusted for *P. aeruginosa*  Model II adjusted for *P. aeruginosa* and absolute BMI  Model III adjusted for *P. aeruginosa*, absolute BMI and age  Model IV adjusted for *P. aeruginosa*, absolute BMI, age and mutation  Model V adjusted for *P. aeruginosa*, absolute BMI, age, mutation and deprivation  Model VI adjusted for *P. aeruginosa*, absolute BMI, age, mutation, deprivation and sex |
| --- | --- |

**Supplementary references**

^1^Patel AR, Patel AR, Singh S, Singh S, Khawaja I. Diagnosing Allergic Bronchopulmonary Aspergillosis: A Review. Cureus. 2019;11:e4550-e

^2^Douglas P, Bakolis I, Fecht D, Pearson C, Leal Sanchez M, Kinnersley R, et al. Respiratory hospital admission risk near large composting facilities. Int J Hyg Environ Health. 2016;219:372-9

^3^Williams B, Douglas P, Roca Barcelo A, Hansell AL, Hayes E. Estimating Aspergillus fumigatus exposure from outdoor composting activities in England between 2005 and 14. Waste Manag. 2019;84:235-44

^4^Roca-Barcelo A, Douglas P, Fecht D, Sterrantino AF, Williams B, Blangiardo M, et al. Risk of respiratory hospital admission associated with modelled concentrations of Aspergillus fumigatus from composting facilities in England. Environ Res. 2020;183:108949.

^5^ONS. Office for National Statistics; National Records of Scotland; Northern Ireland Statistics and Research Agency; UK Data Service. Available from: DOI: <http://dx.doi.org/10.5257/census/aggregate-2011-2> (Accessed on 28 March 2022)
